# The effect of a non-steroidal anti-inflammatory drug on the locomotor activity of reindeer (*Rangifer tarandus tarandus*) on their natural pastures after clamp castration—a pilot study

**DOI:** 10.1186/s13028-025-00802-z

**Published:** 2025-04-09

**Authors:** Hanna Nurmi, Laura Hänninen, Sauli Laaksonen, Anna Valros

**Affiliations:** 1 Department of Production Animal Medicine, Faculty of Veterinary Medicine, University of Helsinki, PL 66 (Agnes Sjöberginkatu 2), 00014 Helsingin Yliopisto, Helsinki, Finland; 2 Research Centre for Animal Welfare, Faculty of Veterinary Medicine, University of Helsinki, PL 66 (Agnes Sjöberginkatu 2), 00014 Helsingin Yliopisto, Helsinki, Finland

**Keywords:** Analgesia, Castration, GPS, Meloxicam, NSAID, Pain, Reindeer, Welfare

## Abstract

**Background:**

During seasonal round-ups, free-grazing reindeer are gathered from natural pastures. Reindeer bulls removed from breeding are clamp castrated, traditionally without analgesia, and then returned to the grazing grounds. The new Finnish Animal Welfare Act requires the use of analgesia in painful procedures. Our earlier studies have shown that a single dose of the non-steroidal anti-inflammatory drug (NSAID) meloxicam may maintain therapeutic plasma concentrations for 2–3 days in reindeer. No studies have been conducted on the effect of meloxicam on the locomotor activity of free-ranging castrated reindeer after castration. We installed GPS collars on 16 male reindeer (at least 5 years old, 130–160 kg), chosen to be castrated as a standard procedure during the round-up held on 5 Oct 2020. Of these, eight were randomly selected to receive approximately 0.5 mg/kg of meloxicam subcutaneously (NSAID group) and eight received no analgesia (TRAD group). The trackers were set to provide location twice per hour with 10 m accuracy. From the GPS data, we calculated the daily distances travelled by the reindeer during the 3 days after castration and analysed the differences between the treatments using a GEE model. Fixed factors were treatment (NSAID or TRAD), days (1–3) and hours, and the interactions between these variables. Our key presumption was that a meloxicam injection can reduce the pain related restless locomotion of newly clamp castrated reindeer.

**Results:**

The overall mean ± SE daily distances travelled by NSAID (n = 8) and TRAD (n = 8) reindeer did not differ (6.60 ± 0.67 km vs. 8.60 ± 1.54 km). However, all reindeer (n = 16) moved more on day 1 than day 3. TRAD reindeer travelled farther than NSAID on day 1 (11.67 ± 2.25 km vs. 7.08 ± 0.61 km, P < 0.05), but no differences were observed on days 2 or 3 due to high variation (10.19 ± 3.87 km vs. 6.59 ± 0.85 km and 5.35 ± 0.39 km vs. 6.17 ± 0.70 m, P > 0.1). NSAID movement remained stable between the days (P > 0.1), while TRAD activity declined (P = 0.002), levelling with NSAID by day 3. Daytime distances exceeded nighttime distances on days 2 and 3, with TRAD showing more disrupted daily rhythms.

**Conclusions:**

Meloxicam may reduce restlessness in newly castrated reindeer, changing postoperative locomotor activity patterns in a way that suggests pain alleviation during the first 2–3 days following clamp castration. Further studies are needed on the use of analgesia and GPS collars for pain monitoring in freely grazing reindeer.

## Background

An estimated 2500–4000 reindeer are clamp castrated annually in the reindeer herding area in northern Finland. Traditionally, the operation has mostly been performed without pain alleviation measures. Free-grazing reindeer herds are gathered from the pastures into seasonal round-up enclosures for counting and slaughtering in late autumn and early winter (October–November). Old and excessive males removed from breeding are clamp castrated and released back to their natural pastures. However, the Animal Welfare Legislation in Finland has been reformed, and from 1 Jan 2024, pain alleviation is mandatory when reindeer are exposed to painful procedures, such as castration [1].

Research on other ruminants indisputably demonstrates that clamp castration is painful [2–5]. Animals move less or stand more after castration and show characteristic pain-related stiff movement or standing postures [6–9]. Moreover, untreated pain manifests as restlessness and increases in locomotor activity [6] and as alterations in resting and sleeping behaviours [10–12]. However, compared to various castration methods, successful clamp castration seems to cause the shortest afterpain period to young bovine calves [13]. The first few hours are the worst [2, 14], and scrotal swelling increases for approximately 2 days, after which symptoms of pain and swelling usually disappear within 15 days [13, 15]. Unlike other ruminants, reindeer are castrated as adults.

Prey animals are generally very stoic to pain, as expressing pain does not give them a biological advantage [8]. In line with this, we have previously reported that reindeer show subtle pain behaviours at the moment of acute pain caused by clamps crushing the tissue [16]. Postoperative castration pain can be treated with nonsteroidal anti-inflammatory drugs (NSAIDs) [17], such as meloxicam; a PGE2 synthesis inhibitor with anti-inflammatory, anti-pyretic, and analgetic properties [18]. We have recently shown that a single dose of meloxicam (0.5 mg/kg intravenously or orally) has the potential to maintain the therapeutic concentration in reindeer plasma, as determined in other species, for up to 3 days [19]. However, no studies exist on how NSAIDs alter reindeer behaviour, such as activity patterns in the wild during the 1 st days after clamp castration.

Reindeer maintain a circadian rhythm throughout the year, with periods of ultradian activity and inactivity alternating over a 24-h period [20, 21]. Dawn and dusk divide reindeer activity into day and night, with daytime activity predominating and the number of activity peaks ranging from 4–6 in winter to 6–9 in summer [22]. Untreated pain can alter the activity patterns of various animal species [12], but no such studies on reindeer have been reported.

In recent years, advanced tracking techniques, such as Global Positioning System (GPS), have proven useful in livestock management and in improving animal welfare [23], and it is currently widely used in reindeer husbandry. The use of GPS on reindeer may minimize human disturbance and influence on the research results of behavioural studies [24, 25], and GPS can be used to track animal movements over large areas, and to determine circadian rhythms and changes in activity under various conditions [23].

We aimed to examine the effect of the NSAID meloxicam on the locomotor activity of newly castrated reindeer over 3 days in winter pastures in northern Savukoski, central-eastern Lapland. Specifically, we compared activity levels between clamp-castrated reindeer treated with analgesia and those without. We hypothesized that castration pain would increase activity in free-grazing reindeer, but that NSAID treatment would reduce this effect, leading to lower activity in treated reindeer compared to untreated controls.

## Methods

**Fig. 1:**
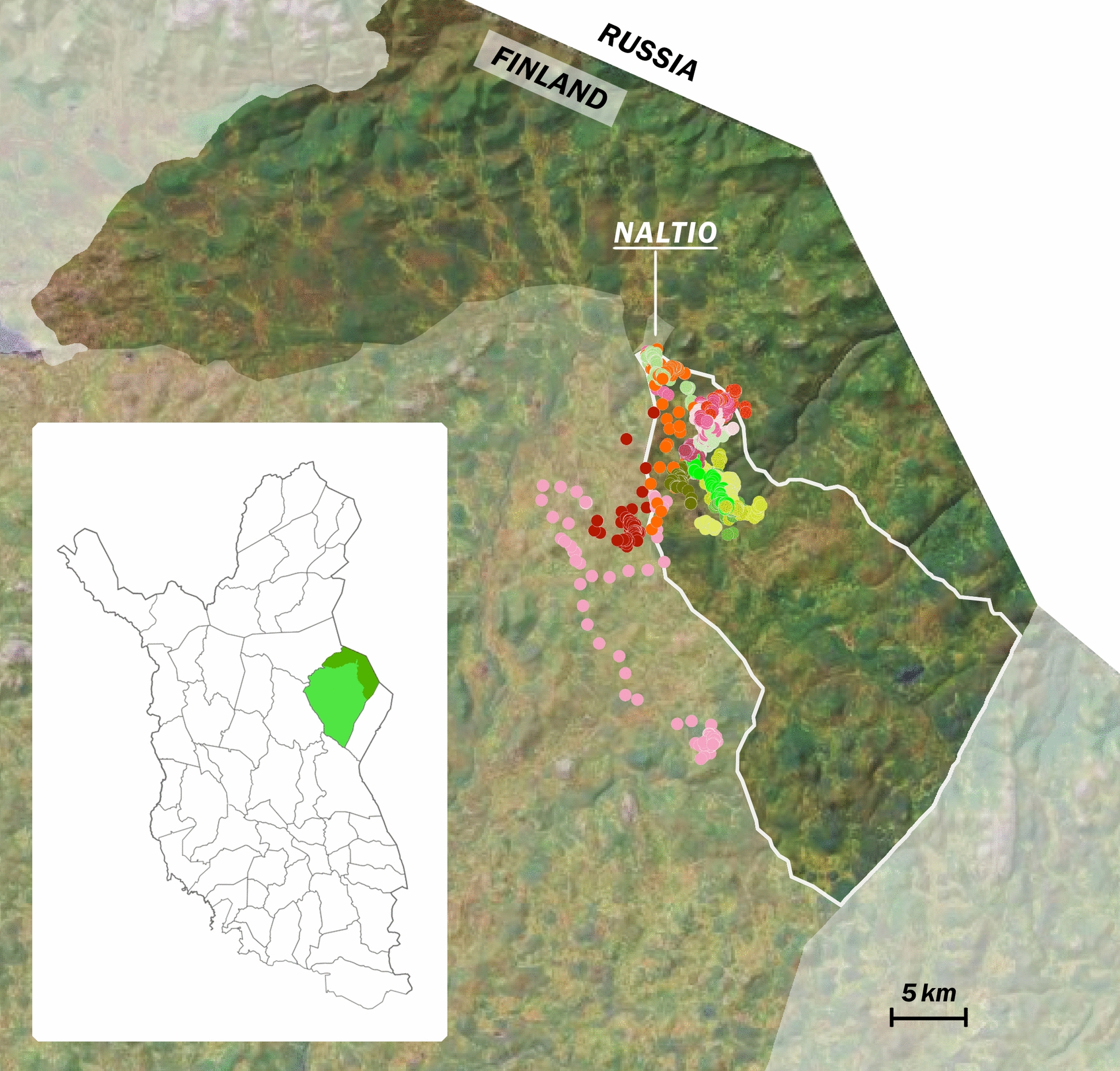
GPS positions of the 16 study reindeer during the 3 days after clamp castration. The left map of the reindeer herding area and cooperatives in northern Finland shows the area of the Kemi-Sompio cooperative in bright and dark green. The dark green area is the winter pasture. The white line indicates the fenced pasture block to which the study reindeer were released from the Naltio round-up. Each point corresponds to the GPS location of one reindeer every half an hour. Individual reindeer are marked with different colours: Red colours belong to the TRAD group and green/yellow to the NSAID treatment. NALTIO is the location of the round-up enclosure for the treatments. Please see www.paliskunnat.fi for more information on the names and location of cooperatives*.*©Flourish, ©OpenMapTiles ©OpenStreetMap contributors

The study was approved by the University of Helsinki Viikki Campus Research Ethics Committee (Statement 16/2020) and the Finnish Medicines Agency (Vetkl-nro 04/2019) and was performed in the Kemin-Sompio co-operative’s one-day round-up in Naltio (67°N 29°E), Savukoski, Finland on 5 October 2020 (Fig. 1).

### Animals and management

We enrolled 16 healthy reindeer that the reindeer owners chose to be castrated as a part of routine reindeer herding at the Kemin–Sompio reindeer co-operative round-up. The randomly chosen reindeer were at least 5 years of age identified from their ear tags, and their estimated weights ranged between 130 and 160 kg. All participating reindeer owners signed an informed consent.

As a standard herding procedure, approximately 1300 reindeer were herded, by helicopter with all-terrain vehicle assistance, from their natural pastures to the Naltio round-up enclosure for 5 days prior to round-up. The round-up area has a large forest enclosure of about 960 hectares, with sufficient space and natural pastures to feed 5000 reindeer, as well as brooks and springs for water supply. During round-up, the reindeer were moved in approximately 100-animal batches to a circular round-up corral in which the animals were identified and counted, their health statuses were checked, the animals for slaughter were separated from the others, unmarked calves were ear-marked, males removed from breeding were castrated, and the breeding reindeer were treated with the anthelmintic drug ivermectin 0.2 mg/kg live weight subcutaneously (Virbamec 10 mg/mL, Virbac, France). After these measures, the reindeer were released into a new, spacious enclosure to await release into the wild after separation. The round-up lasted from approximately 11 AM to 5 PM, after which the animals were released from the enclosures into the wild. The weather was clear, + 5 °C during the treatment day and remained stable during the following 3 days, fluctuating mildly from slightly above 0 °C to a maximum of + 10 °C (measured from three weather observation stations located on the corners of the winter pasture area).

### Study design

The 16 study animals were randomly allocated into two eight-animal groups: The NSAID group (castrated and treated with meloxicam) was treated with a subcutaneous injection of 4 mL Metacam 20 mg/mL inject. (Boehringer Ingelheim Vetmedica GmbH, Germany), and the TRAD (traditional non-medicated castration) group received no analgesia. Animals were caught by hand by 2–3 reindeer herders and immobilized for castration by bringing them down to the ground. Castration was performed with emasculator clamps, crushing the seminal cord for 6–7 s per side. All animals were perioperatively given an ivermectin injection and NSAID-treated animals were injected with meloxicam; all injections were administered subcutaneously on the neck before the animals were allowed to stand up.

After the round up the study reindeer were released to the 430-square-kilometer section of the fenced winter pasture area, characterized by a mixture of differently aged, dry lichen (Cladina spp.)-dominated pine forests, semidry pine and spruce forests, mixed forests, as well as open and wooded bogs. Old-growth coniferous forests are predominantly found in the conservation areas, located in the northwest and southeast parts of the section. However, the majority of this pasture section consists of commercial forestry areas, where felling sites, sapling stands, and young forests dominate. Additionally, there are valleys with riverbanks and meadows populated by deciduous trees. The highest stony hills in the area range from 360 to 420 m above sea level (data derived from Finnish Environment Institute Syke Liiteri map material bank). The reindeer in the study were born and grazed entirely on these remote pastures, making the area’s food sources and locations familiar to them. A few study animals wandered out of the fenced area, probably due to breakdown or openings of the fences (Fig. 1).

### Registering and data handling

To follow the location and acquire data on the locomotor activity of the castrated reindeer, each study animal was equipped with an Ultracom GPS tracker, with an elastic collar belt designed for male reindeer (Tracker Ltd, Oulu, Finland), when brought down for castration. The trackers were pre-set to provide locations twice an hour for three weeks. GPS tracker data was transferred into geographical map points. The distances between two sequential measurement points were analysed using the Spherical Law of Cosines formula calculating the great-circle distance between two points on the Earth’s spherical surface, given their latitude and longitude coordinates:

$$ACOS\left( {COS\left( {RADIANS\left( {90 - L1} \right)} \right) \, *COS\left( {RADIANS\left( {90 - L2} \right)} \right) \, + SIN\left( {RADIANS\left( {90 - L1} \right)} \right) \, *SIN\left( {RADIANS\left( {90 - L2} \right)} \right) \, *COS\left( {RADIANS\left( {D1 - D2} \right)} \right)} \right) \, *6371,$$

in which L1 = latitude 1, L2 = latitude 2, D1 = longitude 1, and D2 = longitude 2.

Three of the GPSs were out of satellite reach for 1–2 h during the first day and for two h on days 2 and 3. Four of the reindeer had 2–12 h of missing data on days 4 to 6, and on day 7 already nine reindeer were out of reach. Three GPS collars were dropped during the 3-week follow-up period. With only 16 animals, we aimed to retain all data points and avoid listwise deletion, not to loose animals from the study. Therefore, we used a Bayesian statistical method for imputing the missing data [26]. Only the three first days were included in the final analyses.

### Statistical analysis

We calculated the average daily and hourly distances travelled by reindeer after castration and the mean hourly distances travelled during daytime (7–17) and night-time (17–7) h. As the data did not follow a normal distribution, we analysed the differences between the treatments (NSAID and TRAD) with three separate models using the Generalized Estimating Equations (GEE), which is suitable for processing repeated measures data when the data distribution is non-normal [27]. For the model analysing the mean daily distances travelled (Model 1), the explanatory factors included the days after castration (1–3), the treatments (TRAD and NSAID), and their interactions. The model that analysed the differences in day-night activity had treatments, days, and day–night times, along with their two-way interactions, as factors (Model 2). Accordingly, the factors used in the model analysing differences in hourly travelled distances over 3 days were treatments (TRAD and NSAID), days (1–3), daily h (24 h), and their interactions (Model 3). In all models, we employed a log-link function, utilized unstructured correlation structures, employed robust estimators for the covariance matrix, and applied Fisher parameter estimation for maximum likelihood estimates. Given the nature of GEE, explicit degrees of freedom for the model cannot be provided.

The analyses were performed utilizing IBM SPSS Statistics (version 29.0, Armonk, NY, USA), and significance was determined at a threshold of P < 0.05.

## Results

### Model 1

Overall, the reindeer (n = 16) travelled 7.53 ± 0.75 kms per day during the 3 days. The variation was high, and no significant differences (P > 0.1) were observed between the NSAID (n = 8) and TRAD (n = 8) reindeer in the mean ± SE overall distances travelled daily during the 3 days (6.60 ± 0.57 km vs. 8.60 ± 1.54 km, respectively). The study reindeer moved mainly separately from each other but within the same geographical area over the three measurement days (Fig. 1).

**Fig. 2:**
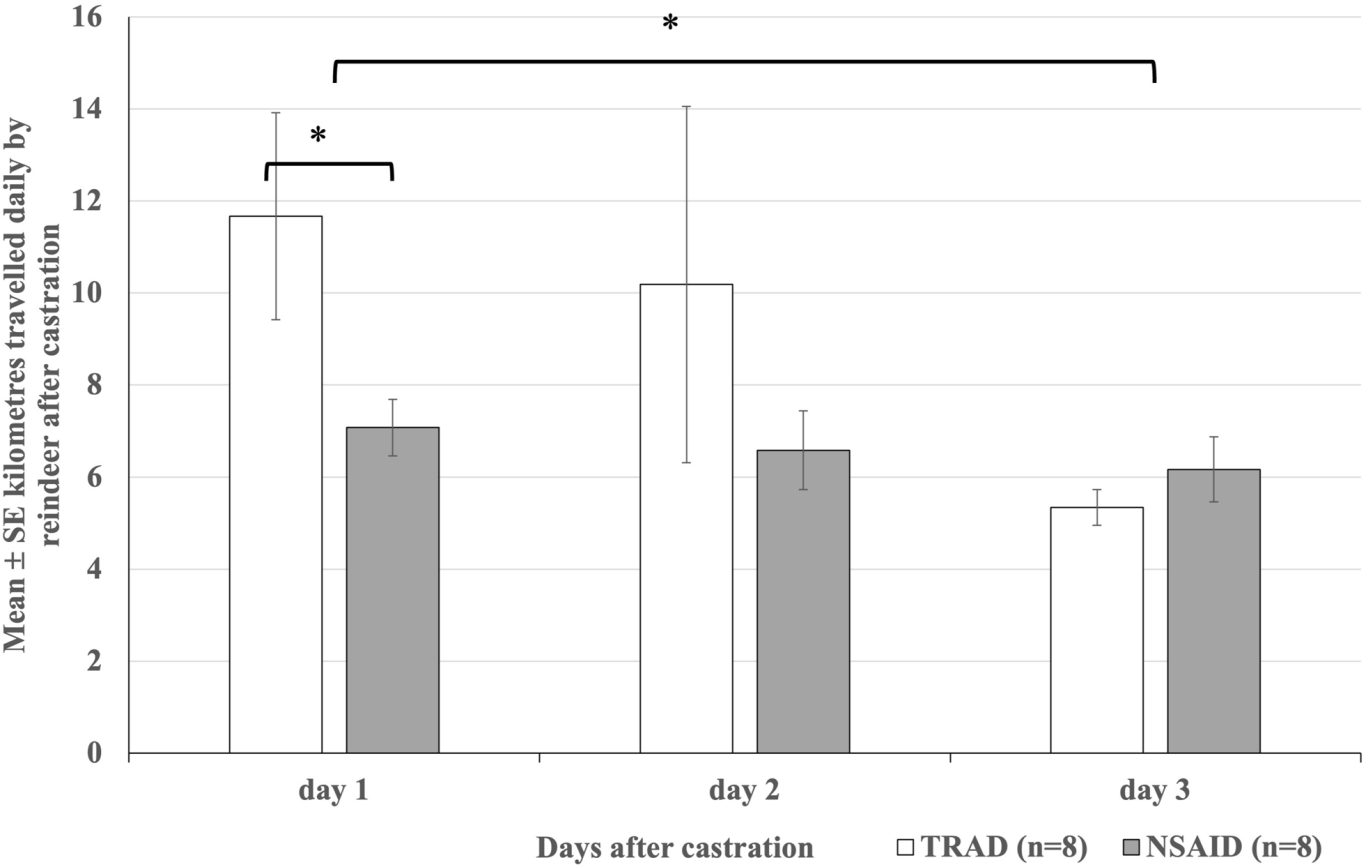
The overall mean ± SE kilometres travelled by the newly clamp-castrated reindeer in the natural pastures. NSAID reindeer (n = 8) were injected with 0.5 mg/kg meloxicam subcutaneously and TRAD reindeer (n = 8) were castrated without analgesia. The whiskers and asterisks mark statistically significant differences tested with GEE model

However, the days differed from each other (P < 0.001): Reindeer (n = 16) travelled longer distances on the first day as compared to the third day after castration (9.09 ± 0.96 km vs. 5.75 ± 0.39 km, P < 0.001), while distances travelled on the second day (8.20 ± 1.64 km) did not differ from either the first or the third day (P > 0.1). Moreover, we found a significant interaction between treatments and days (P = 0.01): TRAD reindeer (n = 8) travelled longer distances than NSAID reindeer (n = 8) during the first day after castration (11.67 ± 2.25 km vs. 7.08 ± 0.61 km, P < 0.05), with no differences observed on the second and third day (10.19 ± 3.87 km vs. 6.59 ± 0.85 km and 5.35 ± 0.39 km vs. 6.17 ± 0.70 m, P > 0.1, respectively). Furthermore, distances travelled by NSAID reindeer did not differ between the days (P > 0.1), but the activity of TRAD reindeer decreased over 3 days (P = 0.002), so that that the originally higher activity of reindeer in the TRAD group at the beginning of the observation period decreased to the same level as in the NSAID group on the third observation day (Fig. 2).

### Model 2

The total hourly daytime and night-time distances travelled by reindeer (n = 16) differed (P = 0.005); the reindeer travelled shorter distances during night-time than during daytime (271.89 ± 24.88 m vs. 360.26 ± 46.06 m, P = 0.02). We observed no overall differences between treatments (P > 0.1). However, there was a significant interaction between days and treatments (P = 0.03), respective to Fig. 2. On day 1, TRAD reindeer travelled the longest distance, which differed significantly from TRAD on day 3 (476.15 ± 89.76 m vs. 226.77 ± 18.71 m, P = 0.002). TRAD on day 1 also tended to differ from NSAID on day 1 (298.2 ± 28.3 m, P = 0.06). Additionally, TRAD on day 1 travelled significantly farther than NSAID on days 2 and 3 (264.07 ± 33.73 m and 251.65 ± 30.3 m, P = 0.03 for both).

**Fig. 3:**
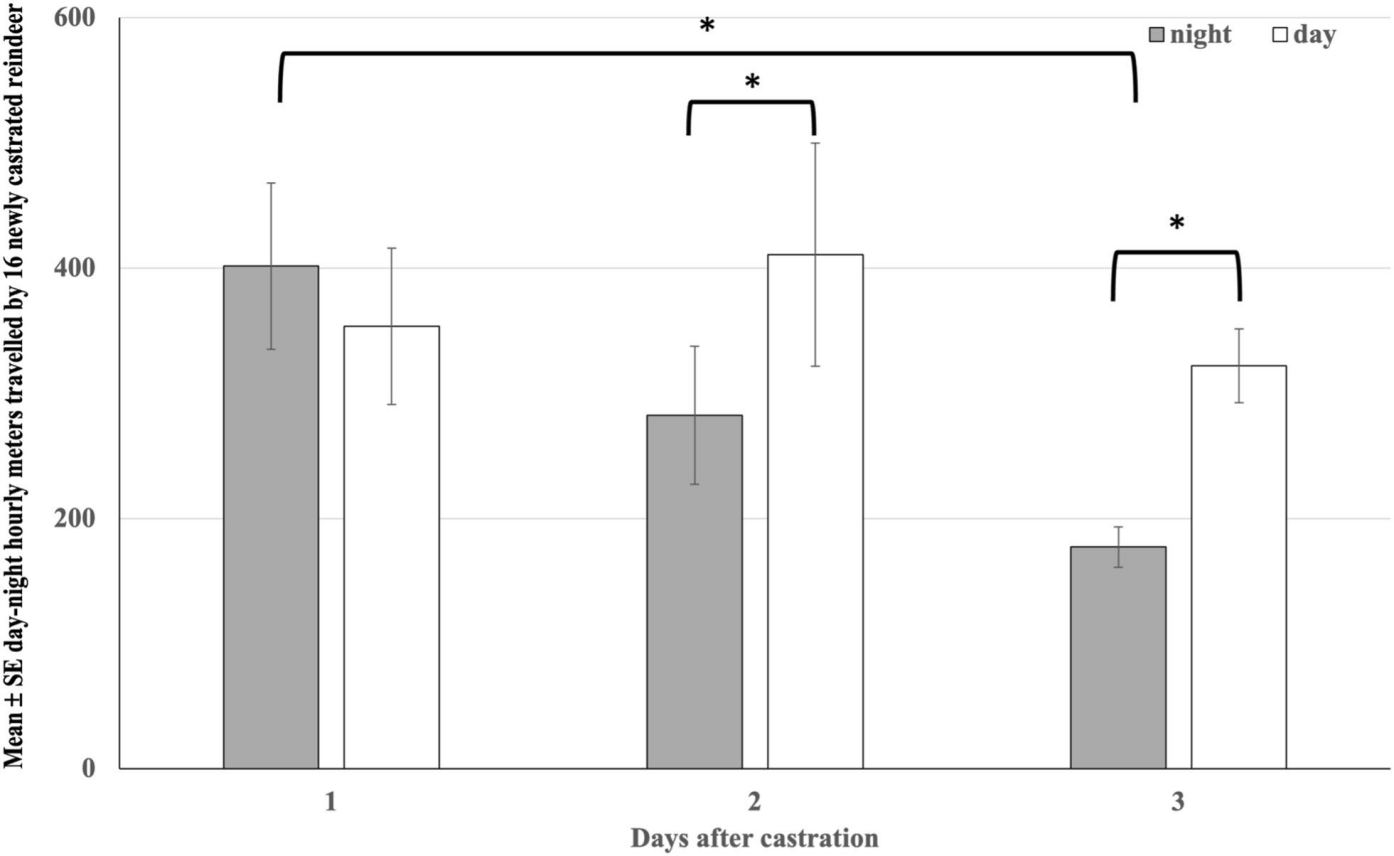
Mean ± SE day–night hourly metres travelled by newly castrated reindeer in natural pastures. NSAID (n = 8) was injected with 0.5 mg/kg meloxicam subcutaneously and TRAD (n = 8) was castrated without analgesia. The study reindeer were followed with GPS for the first 3 days after clamp castration. Statistically significant differences tested with GEE model are marked with whiskers and asterisks

There was also an interaction between the days and the day-night phases (P = 0.04): The total distances reindeer (n = 16) travelled by day and by night did not differ on the first day (P > 0.1), but longer daytime distances were travelled on days 2 and 3 than during respective nights (P < 0.05 for both). The travelled daytime distances remained steady (P > 0.1), but the night-time distances travelled decreased between first and third night (P < 0.001) (Fig. 3).

### Model 3

**Fig. 4:**
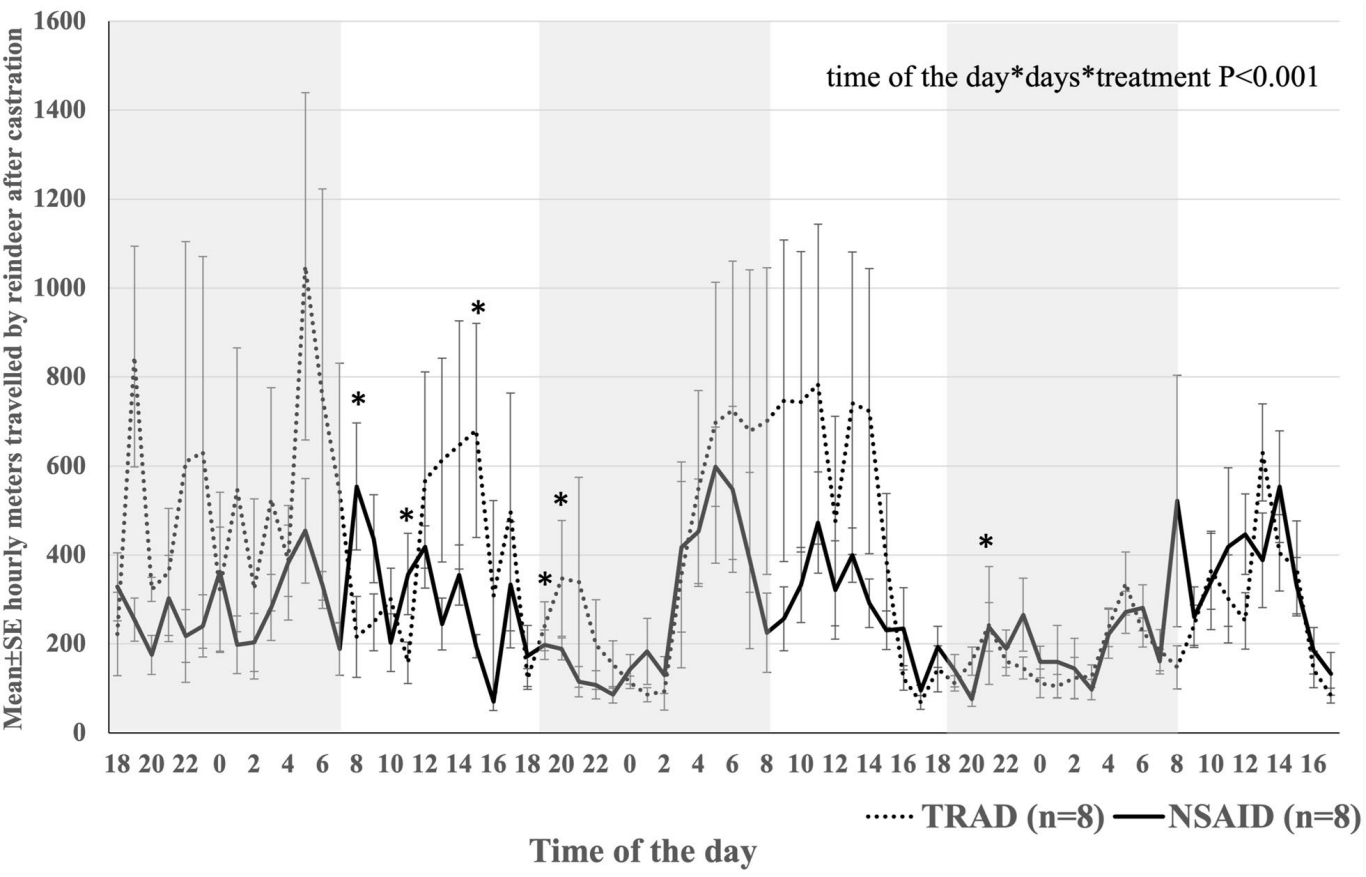
The overall mean ± SE hourly metres travelled by the newly castrated reindeer in natural pastures. The NSAID group (n = 8) was injected with 0.5 mg/kg meloxicam subcutaneously and the TRAD group (n = 8) was castrated without analgesia. The study reindeer were followed with GPS for the first 3 days after clamp castration. The blue shading indicates night-time h. Statistically significant differences tested with GEE model are marked with asterisks

The overall hourly distance travelled by the reindeer (n = 16) was 272.12 ± 20.72 m during the 3 days, which did not differ statistically between TRAD (n = 8) and NSAID (n = 8) reindeer (302.31 ± 39.91 m and 244.94 ± 18.61 m, P > 0.1, respectively). However, we found differences between the days (P < 0.001); overall, reindeer (n = 16) tended to travel longer hourly distances during the first day compared to the second day (345.78 ± 31.38 m vs. 276.39 ± 39.44, P = 0.05) and travelled shorter distances on the third day (210.85 ± 13.51 m) than on the first day (P < 0.001). There was an interaction between days and treatments (P = 0.01) for the hourly distances travelled, and moreover, significant interactions were observed between treatments, days and h (P < 0.001): Overall, the reindeer (n = 16) travelled the longest hourly distances during daytime h (6 AM to 3 PM). The daily rhythm was broken by the more restless TRAD group (n = 8) with a higher locomotor activity during the first day of the observation period (Fig. 4). During the start of the first day after the restless first night, the NSAID group had a higher activity peak than the TRAD group between 8–11 AM, otherwise TRAD reindeer travelled longer hourly distances, especially between 3–8 PM (P < 0.05 for all). However, the variation was high in TRAD (Fig. 4).

## Discussion

Here, we show for the first time that untreated pain increases the movement of castrated reindeer in the wild. The subcutaneously administered NSAID meloxicam reduced locomotor activity of castrated reindeer, which indicates a reduction of pain-related restlessness during the first 2 days after castration compared to reindeer castrated in the similar traditional way without NSAIDs. This is shown in the stable NSAID movement during the 3 days, while the originally higher activity of TRAD declined, levelling with NSAID by day 3. The overall day-night activity difference, i.e. reindeer being more active during daytime, disappeared during the first day after the clamp castration but returned from the second day onwards. GPS collars are promising devices for monitoring pain behaviour related to locomotor activity in free-ranging reindeer.

Immediately after the round-up and castration, reindeer from both treatments exhibited activity during the first night and lost their normal diurnal activity rhythm. The loss of diurnal rhythm may be attributed to several factors in addition to castration and castration pain, including the stress of gathering and round-up, short-term irritation caused by the ivermectin injection, as reported by the manufacturer [28], and/or the potential tissue irritation caused by subcutaneous meloxicam, as suspected in our previous study [16]. Following the first night, the reindeer in both groups resumed a clear diurnal activity pattern, consistent with previous observations [22].

We found that clamp-castrated TRAD reindeer were particularly active during 2 days after castration, and their activity decreased to the same level as NSAID reindeer on the third day after castration. After the first restless night, the TRAD group moved less than the NSAID group the following morning. Overall, the NSAID group maintained a more consistent level of movement over the first 3 days. Our results are in line with previous findings showing that post-castration pain is the worst during the first 2 days after the procedure [2, 13, 14]. The subcutaneously administered meloxicam appeared to decrease reindeer activity compared to untreated reindeer, especially during the first 2 days. Although only the intravenous and oral routes of administration have been studied in reindeer thus far, our earlier studies have shown that serum concentrations in reindeer remained at the presumed therapeutic level for 36–72 h regardless of the route of administration [19]. Also, in goats and sheep, both subcutaneous and intramuscular injections of meloxicam achieved rather rapid high plasma concentrations at therapeutic levels for up to 48 h [29, 30].

Notably, due to our study being a pilot experiment, we lacked a non-castrated control group. Moreover, the rut was still on and fitting GPS collars on fighting bulls would have created an animal welfare concern, and the rutting behavior of non-castrated males would have probably confused comparisons of behavior between the non-castrated and castrated males. Increased activity after a painful procedure could either be a positive or a negative sign [31]. However, reindeer in general is a rather vigilant and flight-reactive species [32–34] and thus increased activity in the grazing lands because of pain is not unexpected and in line with other studies on pain and activity [35]. Moreover, comparisons with GPS data from the Natural Resources Institute Finland (LUKE) female reindeer, collected in the same area and season, validate our NSAID group findings. Although not directly comparable, the 4-km distances travelled per day by the LUKE reindeer [36] were more in-line with our NSAID reindeer. Our study reindeer mainly moved separately. Social behaviours play less of a role during the autumn rut with males; even though they tend to form separate groups during summer, during rut they do not tolerate other males [37].

We faced some challenges in our data collection, dealing with a semi-domesticated species whose individuals are prone to ridding themselves of any foreign objects attached to them. Moreover, our study animals were grazing in the natural pastures close to the border of Russia where mobile network coverage is poor at some places. Ultracom tracker uses the mobile network to send the data to the server. Several blind spots were observed when collecting GPS signals and reindeer locations, and these blind spots increased the further our study animals grazed from the round-up enclosure. Therefore, only the first 3 days were analysed. Despite the prominent variation and missing data, the current analysis was sensitive and sufficient for answering our research question, showing that reindeer appear to benefit from NSAIDs administered at the time of clamp castration.

## Conclusions

Our study indicates that analgesia with meloxicam improves the welfare of newly clamp-castrated reindeer. As a single dose of meloxicam reduced post-castration locomotor activity, a potential sign of reduced pain-related restlessness, our results indicate that meloxicam alleviated post-castration pain and discomfort. This was supported by our findings that meloxicam-treated reindeer exhibited less restless locomotion and travelled less after castration compared to those castrated without analgesia.

Further studies are needed on the efficacy, toxicity, therapeutic levels, and optimal routes of administration of various NSAIDs to reindeer, to establish the best practice in using analgesia in reindeer castration.

## Data Availability

The datasets used and analysed during the current study are available from the corresponding author on reasonable request.
